# Implementation Intention for Initiating Intuitive Eating and Active Embodiment in Obese Patients Using a Smartphone Application

**DOI:** 10.3389/fpsyt.2017.00243

**Published:** 2017-11-21

**Authors:** Damien Brevers, Anne Rogiers, Alexis Defontaine, Guy Cheron, Anne-Marie Clarinval, Jennifer Foucart, Anne Bouchez, Véronique Bolly, Laura Tsartsafloudakis, Pénélope Jottrand, Pierre Minner, Antoine Bechara, Charles Kornreich, Paul Verbanck

**Affiliations:** ^1^Laboratory of Psychological Medicine and Addictology, Faculty of Medicine, Université Libre de Bruxelles, Brussels, Belgium; ^2^Research in Psychology Applied to Motor Learning, Faculty of Motor Sciences, Université Libre de Bruxelles, Brussels, Belgium; ^3^Laboratory of Neurophysiology and Movement Biomechanics, Faculty of Motor Sciences, Université Libre de Bruxelles, Brussels, Belgium; ^4^Department of Psychology, Brain and Creativity Institute, University of Southern California, Los Angeles, CA, United States

**Keywords:** smartphone application, obesity, health at every size, intuitive eating, intuitive exercise, implementation intention

## Abstract

This article describes a study protocol, which aims to explore and describe the feasibility of a mobile-phone application for initiating intuitive eating and intuitive exercising in patients who are following an ambulatory treatment for obesity. Intuitive eating refers to one’s ability to make food choices based on one’s awareness of his/her body’s response. Intuitive exercising encourages people in finding enjoyable ways of being physically active. These two components will be trained using an implementation intention procedure, that is, behavioral plans that aim at creating a strong link between a specified situation and a response. We aim to recruit up to 80 overweight and obese patients over a period of 2 years. The smartphone application will be assessed on the basis of (i) data obtained through a 4-week use period, (ii) self-report measures taken before and after the use of the mobile application, and (iii) feedbacks from participants after the use of the mobile application. This pilot study will allow us to better understand the applicability of the use of mobile application within ambulatory treatment settings, and to adapt the design of the app necessary for building cross-sectional studies investigating its efficacy.

## Introduction

Since the early 2000s, the Health at Every Size approach [HAES (1)] has challenged traditional public health views stating that obesity and overweight are unilaterally linked to major negative health consequences (2), and that the effective means to assist obese and overweight individuals is to combine a reduction in caloric intake with an increased physical activity to reduce their weight and their risk for chronic illness [e.g., Ref. (3, 4)]. The HAES approach contrasts with this view by encouraging embodiment-like physical activity and healthy nutrition, while respecting individual’s own rhythm, regardless of weight status.

Specifically, the HAES approach supports “intuitive eating” and “intuitive exercise.” Intuitive eating (also known in the literature as “attuned eating” or “mindful eating”) encourages the awareness of body’s response to food and the learning on how to make food choices that reflect one’s own “body knowledge” (5). This process allows people to make connections between what they eat and how they feel [e.g., mood, satiety, ease of bowel movements, and comfort eating (5–11)]. Intuitive exercise [also referred to as “active embodiment” (5)] encourages people to find enjoyable ways of being physically active, independent of weight loss and of explicit guidelines for frequency and intensity of exercise (5).

As a whole, HAES-based interventions have been shown to improve or maintain behavioral, psychological, physiological, and clinical outcomes, including weight loss (1, 5, 12–14). Importantly, even in the case of weight regain, maintained behavioral practices, such as those produced in HAES interventions, have sustained health benefits [e.g., better blood pressure and lipids, higher energy expenditure, less susceptibility to hunger, higher self-esteem, lower depression, and better self-perception of body image (1, 13, 15–19)]. One explanation for these observed outcomes in case of weight regain is that, by not using weight changes as a marker for health, individuals may be less discouraged by weight stabilization or gain (20, 21).

There is considerable evidence that intuitive eating can be learned [e.g., Ref. (1, 13, 22–26)]. Nevertheless, coming to eat intuitively is a challenging and gradual process, which requires replacing old food habits by new ones (5). Accordingly, HAES-based interventions are usually offered by professionals from health services and include psychoeducation and behavioral therapy sessions [e.g., Ref. (17, 23)].

In the present research project, we advance that the implementation of intention interventions in health behavior change could be used as a promising and complementary established approach for stimulating intuitive eating and active embodiment by bridging the gap between intentions to perform a particular behavior and the actual behavioral change (27–30). Implementation intentions are behavioral strategies that follow an “if–then” structure, which aims to create a strong link between a specific situation and a response. Implementation intentions will allow people to select the appropriate response when confronted to a specified situation [e.g., Ref. (31, 32)]. These “if–then” strategies will enhance health behavior by linking a critical situation (e.g., “IF I am taking the stairs instead of the elevator”) with an appropriate response (e.g., “… THEN I will enjoy the feeling of having my body active”). Overall, implementation intention interventions have been shown to be effective in promoting behavior changes, including physical activity, eating habits, smoking, alcohol consumption, rehabilitation from injury, sun-screen use, cancer screening behaviors, contraception use, and dental health behaviors [for a review see Hagger and Luszczynska (29)].

This study protocol will thus examine the impact of implementation intentions (i.e., an established intervention procedure) as an initial intervention for initiating intuitive eating and active embodiment (i.e., an established intervention approach) in overweight and obese patients. A couple of studies have already examined the effect of implementation intention and goal planning intervention in obesity (33–36). It has been shown that the development of implementation intentions to adhere to a weight-loss program (e.g., “I will try to lose 1 kg by consuming 3–4 portions of fruit”) can achieve greater weight reduction (34, 35). Two ongoing studies are currently examining the impact of implementation intention on physical exercise, energy/calorie intake, and eating strategies (33, 36). Nevertheless, while weight loss could trigger positive (short-term) outcomes, it is usually followed by weight regain [e.g., Ref. (37–40); see also Ref. (41)], which could be detrimental on both the physical [e.g., the weight regained does not replace bone mass or lean mass lost during weight loss (42, 43)] and the mental health of the individual [stress, depression, dissatisfaction toward weight loss, and stigmatization (20, 44–47)]. These considerations highlight the need for more evidence on the usefulness of implementation intentions in obesity, such as using interventions that focus primarily on intuitive eating/exercise rather than weight loss.

Another innovative aspect of this study is that the implementation intention intervention will be undertaken through the use of a mobile-phone application. A main advantage of using mobile devices is its ease of use, as compared with paper tools (48–51): mobile-phone applications have repeatedly been found to improve the completeness and accuracy of patient documentation. Hence, because it allows the direct coding of behavior observations, the use of a mobile app device could provide accurate [e.g., diminished memory bias (52)] and detailed information in the enactment of specific strategies of implementation intention [e.g., frequency, time, subjective experience of efficiency, difficulty, or satisfaction (53)]. Therefore, we reasoned that a mobile-phone application could be used as a promising new tool for enhancing the efficiency of an implementation intention intervention that aims at initiating intuitive eating and active embodiment in individuals who seek help regarding overweight or obesity problems.

## Materials and Equipments

### Participants

Participants will be recruited at the Interdisciplinary Clinic for Obesity (CITO) of the Brugmann University Hospital (Université Libre de Bruxelles, Brussels, Belgium). The CITO unit proposes group therapy sessions in a day clinic setting for patients suffering from to individuals who seek help regarding overweight or obesity problems. The program consists of a twice-monthly stay in the day clinic of the hospital every 2 weeks over 12 sessions during 6 months (6 months in total). A morning session is dedicated to active embodiment (e.g., stretching and hiking), cooking classes and guidance on nutritional habits, provided by mental health professionals (psychologists, nurses, and occupational therapists). The afternoon is dedicated to therapeutic group sessions where patients reflect on how they experienced their last 15 days with regards to their eating and physical activity habits. These discussions are moderated by two certified psychotherapists of the CITO unit within the theoretical framework of mindful eating and intuitive exercise.

We aim to recruit up to 80 participants throughout a 2-year period. This number will allow obtaining a representative sample of patients for this pilot study. Participation will be voluntary and participants will be provided with a study information sheet and consent form. Participants should be at least 18 years old and must own an Android or IOS phone with Internet access.

### Design of the “IF → THEN” Mobile Application

**Figure 1:**
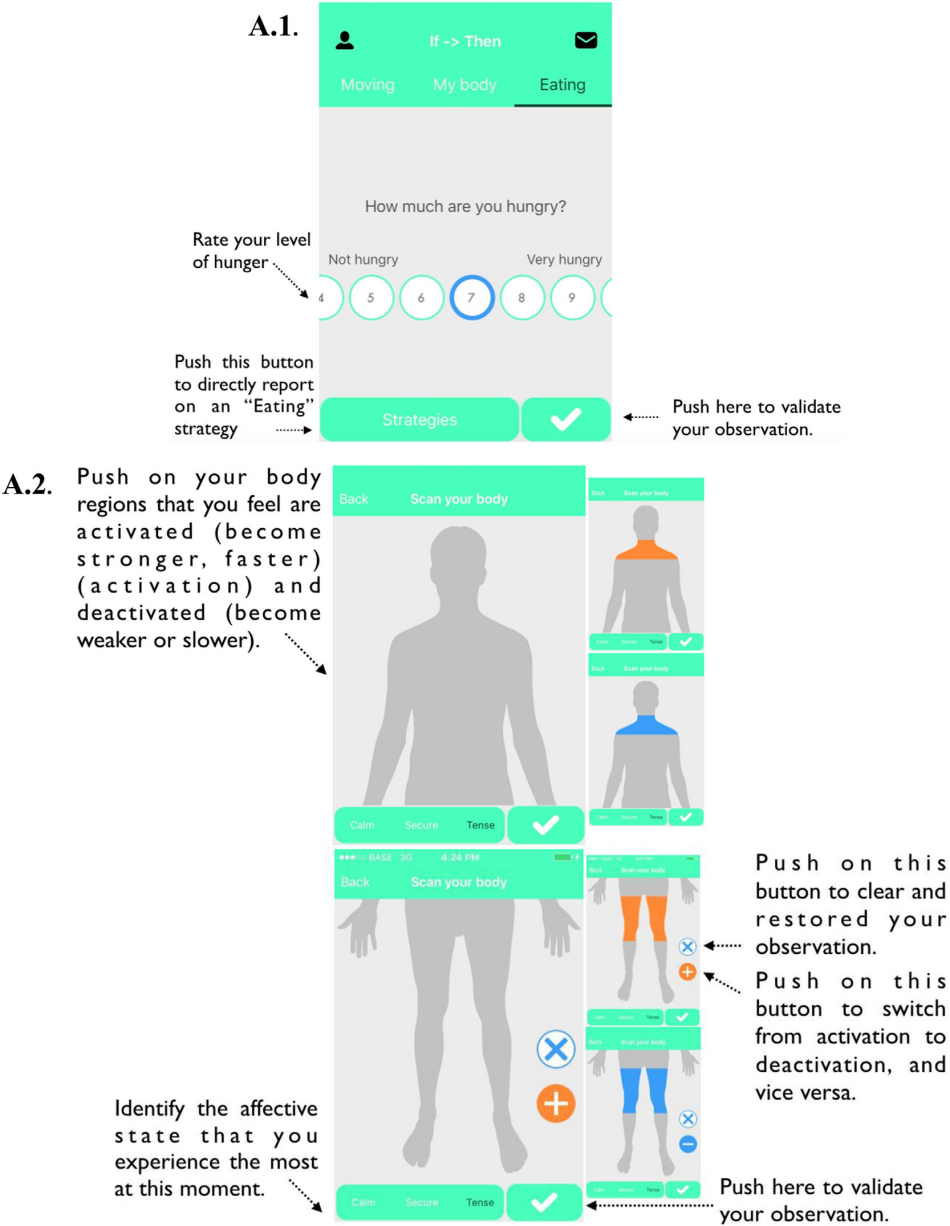

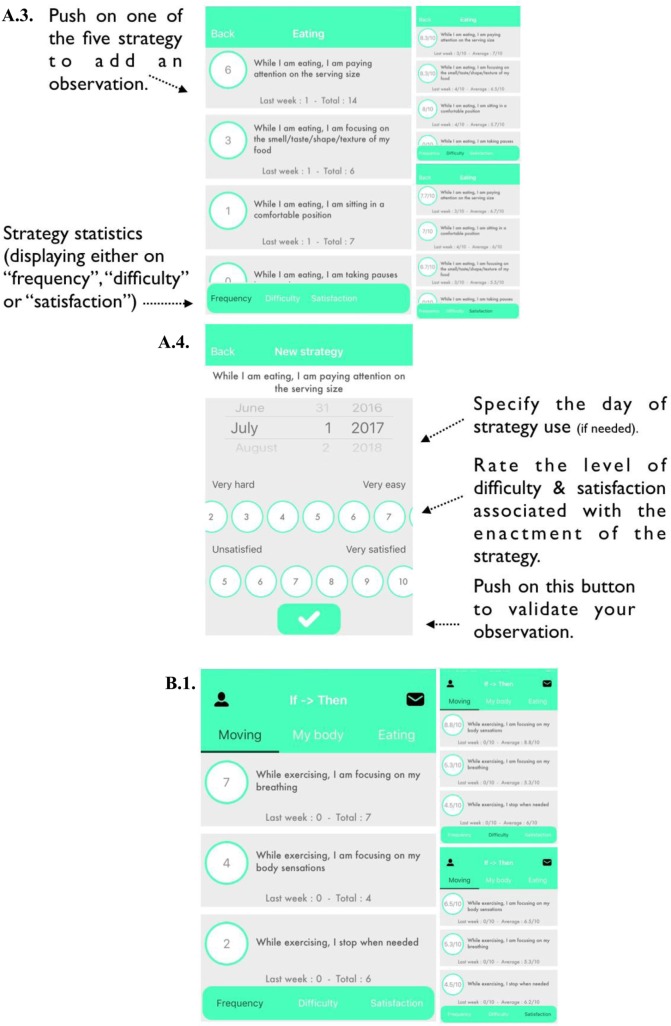

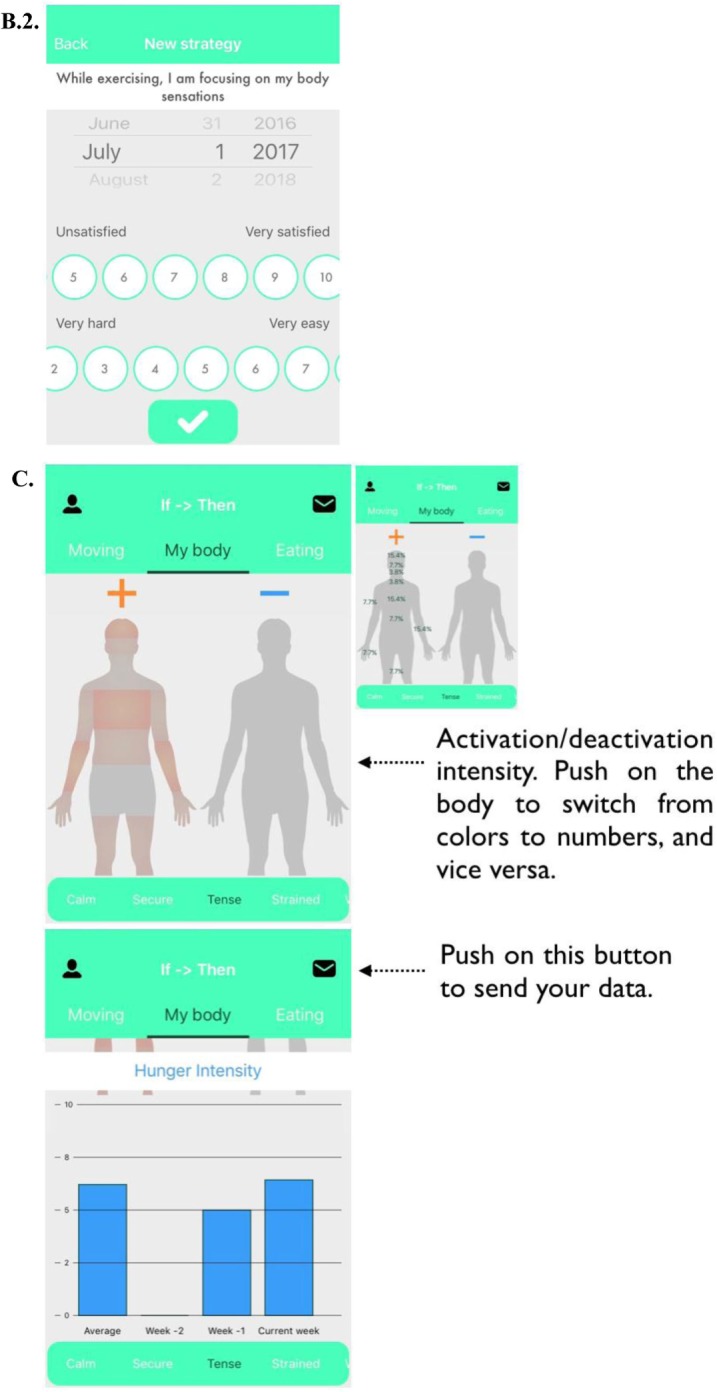
An illustration of the “IF → THEN” smartphone application. **(A1)** After having pushed on “Eating,” participant can either rate the degree of hunger or have a direct access to the implementation intention strategies; **(A2)** after having rated the degree of hunger, the “Scan your body” screen appears; **(A3)** next, the implementation intention strategies appear; **(A4)** after having pushed on a specific strategy, participant has to rate the difficulty and satisfaction associated with the enactment of this strategy; **(B1)** after having pushed on “Moving,” the implementation intention strategies appear; **(B2)** after having pushed on a specific strategy, participant has to rate the difficulty and satisfaction associated with the enactment of this strategy; **(C)** after having pushed on “My body,” participants can have access to descriptive statistics linking the level of hunger associated with each type of affective state, and the topographical observation on body activation/deactivation for each type of affective state.

Sample screen shots of the “IF → THEN” mobile app are demonstrated in Figure 1. The building of the design and structure of the “IF → THEN” mobile app took place between July 2016 and May 2017. It was the result of several multidisciplinary meetings of scientific researchers (Université Libre de Bruxelles) and clinical practitioners (CITO unit). The aim was to create a mobile tool and to select implementation intention strategies that should have the greatest impact and adherence rate in obese patients with regard to mindful eating and embodiment-like physical activity. The “IF → THEN” mobile app includes two main categories of implementation intention strategies: “EATING,” which focuses on mindful eating; and “MOVING,” which focuses on intuitive exercise (i.e., embodiment-like physical activity). Each category includes 5 strategies of implementation intention (i.e., 10 strategies in total; see Table 1 for a listing of the strategy). We decided to use five implementation strategies, as it has been shown that this number leads to the greatest behavior engagement into implementation intention interventions (54).

**Table 1:** Implementation intention strategies that will be proposed to the patient.

	“IF”	“THEN”
EATING	While I am eating	I am sitting in a comfortable position I am paying attention on the serving size I am focusing on the smell/taste/shape/texture of my food I am taking pauses between bites I am assessing my degree of fullness before I decide to take another portion of food
MOVING	While exercising	I am enjoying the surroundings I am focusing on conscious breathing I am focusing on my body sensations I am feeling proud of myself I stop when needed

The EATING category includes an optional step of observation, namely: the recognition of (i) the intensity of hunger, (ii) inner body sensation, and (iii) affective state (see Figure 1A). Participants will be invited to undertaken these steps before eating. Specifically, participant will be first requested to self-report the level of their hunger (on a 10-point scale). Thereafter, they will have to (i) select the bodily regions in which they feel increasing or decreasing (divided in 13 regions-of-interest; see also Figure S1 in Supplementary Material) based on a topographical self-report method (55) and (ii) identify their affective state based on the State-Anxiety Inventory (56): calm, secure, tense, strained, at ease, upset, satisfied, frightened, comfortable, self-confident, nervous, jittery, indecisive, relaxed, content, worried, confused, steady, and pleasant. The goal of this procedure is to reinforce the intuitive eating approach, which encourages the individual to identify the intensity of hunger while recognizing the inner body sensations emerging from affective feelings (5, 20).

After these aforementioned (non-mandatory) steps of observation (i.e., the intensity of hunger, inner body sensation, and affective state), participants will have the possibility to report on the use of an EATING strategy. Participants will also be able to report directly after they used a strategy without going through the first steps of observation (see Figure 1A). Participants will report on each strategy use, by indicating (i) the level of difficulty associated with the enactment of the strategy (on a 10-point scale); and (ii) the level of satisfaction associated with the enactment of the strategy (on a 10-point scale). Participant will be instructed to report on the strategy after finishing eating, not to interfere with its enactment and with the action of eating. Participants will be able to use multiple strategies simultaneously or consecutively: e.g., when the participant is intermittently focusing on the taste of the food and on the size of the serving, he/she should report on the use of each strategy and on the associated levels of difficulty and satisfaction.

In the MOVING category, the participants will be simply requested to “push” on the implementation intention strategy that they just have been undertaken (see Figure 1B). Thereafter, the participants will be asked (i) to rate the level of difficulty associated with the enactment of the strategy (on a 10-point scale); (ii) to rate the level of satisfaction associated with the enactment of the strategy (on a 10-point scale); and (iii) to validate their observation. Participants will be instructed to report on the strategy after their physical exercise not to interfere with the activity. Similarly as in the EATING category, participants will be able to use multiple strategies simultaneously or consecutively (e.g., when a participant has intermittently focused on bodily sensation and on his/her environment during his/her exercise), he/she will have to report on the use of each strategy and on the associated levels of difficulty and satisfaction.

For both the EATING and MOVING categories, participants will have access to numerical information related to their observations (see Figures 1A,B). Specifically, participants will be able to see for each strategy the frequency (i.e., the number of time that a strategy is used), the average level of difficulty (i.e., calculated across each strategy use), and the average level of satisfaction (i.e., calculated across each strategy use). Participants will also have the opportunity to view these numbers according to a specific time interval (i.e., last week, total). Through the function “MY BODY,” they will view the level of hunger associated with each type of affective state, and the topographical observation on body activation/deactivation for each type of affective state (see Figure 1C).

## Stepwise Procedures

### Presentation of the “IF → THEN” Mobile App to the Participants

The study protocol will be explained in detail to the patients during the first day of the CITO therapy. Patients will receive information on (i) implementation intention strategies on the MOVING and EATING categories and (ii) how to use the app in his/her daily life. Patients who accept to participate to the study will be asked to sign the informed consent. All patients will receive the standard of care of the psychotherapeutic CITO program. Patients accepting to participate will have the possibility to use the app additionally to the standard care. The use of the app will not be discussed during the therapeutic sessions.

### Individualized Tutorial on the IF → THEN App

This individualized tutorial session will be organized after 2 weeks after the presentation of the app. During this session, a member of our research group will give detailed instructions to the participant on how to use the app in his/her daily life. To closely approximate real-world conditions, there will be no specific instructions given for frequency of use of the app other than to report each use of an implementation intention strategy.

Then, participant’s subjective motivation to use the mobile application will be assessed by asking the following question: “Are you motivated to use this app for 4 weeks?” (on a 7-points scale, ranging from “non-motivated” to “highly motivated”). Next, participant will complete the French version of the Intuitive Eating Scale-2 (57) and the Intuitive Exercise Scale (58). The Intuitive Eating scale is a 18-items self-reported questionnaire (5-point Likert scale; ranging from “Strongly Disagree” to “Strongly Agree”) that includes three dimensions: (i) eating for physical rather than emotional reasons (eight items; e.g., “I find other ways to cope with stress and anxiety than by eating”); (ii) reliance on hunger and satiety cues (six items; e.g., “I trust my body to tell me when to eat”), and (iii) unconditional permission to eat (four items; e.g., “I do not follow eating rules or dieting plans that dictate what, when, and/or how much to eat.”). The Intuitive Exercise Scale is a 14-items self-reported questionnaire (5-point Likert scale; ranging from “Strongly Disagree” to “Strongly Agree”) that includes four dimensions: emotional exercise (5 items; e.g., “I use exercise to help soothe my negative emotion”), body trust (3 items; “I trust my body to tell me how much exercise to do”), exercise rigidity (3 items; “I engage in a variety of different types of exercise”), and mindful exercise (3 items; “When my body feels tired, I stop exercising”). The Intuitive Exercise Scale will be translated into French by using back translation. Participants will also complete the French version of the 13-item Brief Self-Control Scale [BSCS (59, 60)], a widely used measure of trait self-control. Items (e.g., “I am good at resisting temptation”) are endorsed on a 5-point scale, where 1 = *not at all like me* and 5 = *very much like me*. This measure was added to the protocol on the basis of previous studies which have shown that high trait self-control predicts both positive health behaviors and success in weight loss [e.g., Ref. (61)].

After having completed these self-report measures, participants will be asked to use the app for 4 weeks and a feedback session will be scheduled.

### Data Collection and Mobile App Rating

This session will occur 4 weeks after the individualized tutorial session. First, the participant will be asked to send the data from the “IF → THEN” app by pushing on the “send” icon (see Figure 1C). This procedure will allow to save the data (under.xls file format) to a secure server. To protect against loss of confidentiality, all data will be identified by a unique numeric ID code. The list linking the participants’ ID codes with their names will be stored in a password-protected file on an internal server, accessible only to the experimenters and selected project staff. Secondly, the participant will complete the Intuitive Eating Scale-2 and the Intuitive Exercise Scale. Finally, the participant will be invited to fill in an anonymous feedback form on the level of (dis)satisfaction with their overall app user experience and with regard to the MOVING, EATING, and MYBODY sections.

### Follow-up

After completion of the CITO program (i.e., 6 months after the tutorial session), participants will be invited to fill-in the Intuitive Eating Scale-2 and the Intuitive Exercise Scale.

### Ethics Approval and Current Status of Project

The study protocol has been approved by the CHU-Brugmann University Hospital Institutional Review Board (REF: B077201732743/I/U). The IF → THEN mobile application is available (on both Android and IOS smartphone) under request to dbrevers@ulb.ac.be. Data collection is planned to start in October 2017 until October 2019.

## Anticipated Results

### Main Goals

The aim of this study is to explore and to describe the use of a mobile-phone application, using “IF → THEN” strategies to initiate intuitive eating and intuitive exercise in overweight and obese patients. In other words, this exploratory study is to gain some insight on the mechanisms attached to an implementation intention intervention, targeting intuitive eating and embodiment-like activity that are proposed through a mobile app. Therefore, we expect that the information gained from this study will help to collect useful information for improving intuitive eating and intuitive exercise with a mobile application in clinical settings and, ultimately, conducting cross-sectional studies for testing the efficacy of such type of intervention.

### Specific Research Questions and Applicable Data Analyses

#### Is There a Difference in Frequency, Difficulty, and Satisfaction between the Five Strategies of Implementation Intention?

McNemar tests (corrected for multiple comparisons using Bonferroni–Holms) will be used to examine differences in the frequency of use between each five strategies. Repeated measures analyses of variances (ANOVA) will be undertaken with the five types of strategies as within-subject factor, and with scores of difficulty or satisfaction as dependent variables. These analyses will be undertaken separately for the EATING and MOVING categories.

#### Do Frequency, Difficulty, and Satisfaction Attached to Implementation Intention Strategies Differ over Time?

Scores of frequency, difficulty, and satisfaction will be averaged across the five strategies of implementation intention, separately for each week of use (weeks 1, 2, 3, and 4). Reliability for these indices (i.e., frequency, difficulty, and satisfaction) will be estimated with Cronbach’s alpha coefficients. Third, repeated measures ANOVA will be undertaken with weeks (1, 2, 3, and 4) as within-subject factor, with either frequency, mean score of difficulty or mean score of satisfaction as dependent variables.

#### Does Motivation to Use the IF → THEN App and Trait Self-Control Are Associated with Self-Reported Improvements of Intuitive Eating and Intuitive Exercise?

First, scores on the Intuitive Eating Scale-2 and the Intuitive Exercise Scale obtained in step 2 and step 3 will subtracted from the one obtained in step 4 (i.e., step 4 minus step 2; step 4 minus step 3), respectively. Second, Pearson correlation analyses (corrected for multiple comparisons using Bonferroni–Holms) will be undertaken between scores of app motivation use (obtained in step 1), BSCS scores, and the two computed scores of intuitive eating (step 4 minus step 2; step 4 minus step 3) and intuitive exercise (step 4 minus step 2; step 4 minus step 3).

#### Data Obtained from Bodily Sensation Maps and Affective States

Data recorded through the “MY BODY” tool will be examined through frequency and descriptive levels of analyses on bodily sensation maps and affective states.

### Pitfalls, Artifacts, and Troubleshooting

The smartphone application will be assessed during 4 weeks, which is a limited assessment time. However, this length is common for implementation intention interventions (29). This short-time period has also been chosen to minimize missing data and respondent’s time burden, which could have been increased by a prolonged assessment period. The stepwise procedure has also been designed in order for the participants to perceive the app use as an inherent part of their treatment. Indeed, the mobile app focuses on two components (intuitive eating and active embodiment) that are being learned during their group therapy sessions at the CITO unit. Moreover, participants will receive step-by-step information of the participation process. In addition, the mobile app “IF → THEN” was designed in an attractive, and intuitive navigable way, which might further increase participant’s level of engagement. Finally, participant’s motivation to use the app will be assessed in the beginning of the intervention. This should inform us on how intuitive eating and intuitive exercise improved among participants with different levels of engagement toward mobile app use, and further shed light in potential feasibility drawbacks.

## Ethics Statement

The study protocol has been approved by the CHU-Brugmann University Hospital Institutional Review Board (REF: B077201732743/I/U).

## Author Contributions

DB, AR, AD, A-MC, ABouchez, VB, JF, ABechara, CK, LT, PJ, GC, PM, and PV designed the study and wrote the protocol. DB and AR wrote the first draft of the manuscript, and all authors contributed to and have approved the final manuscript.

## Conflict of Interest Statement

The authors declare that the research was conducted in the absence of any commercial or financial relationships that could be construed as a potential conflict of interest.
