# Supplementary material for: Implementation Intention for Initiating Intuitive Eating and Active Embodiment in Obese Patients Using a Smartphone Application
Source: Front Psychiatry. 2017 Nov 21;8:243. doi: 10.3389/fpsyt.2017.00243 (PMC5702316; doi:10.3389/fpsyt.2017.00243)
Supplement: Supplementary file 1 [file data_sheet_1.docx]

***Supplementary Materials***


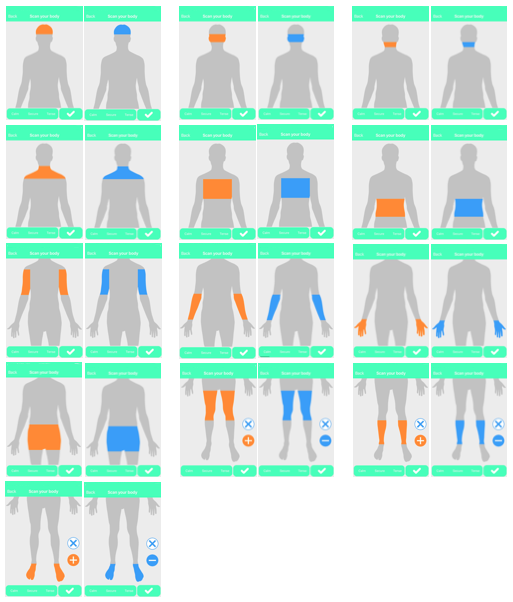


**Figure S1.** Graphical depiction of the body regions-of-interest used in the “IF->THEN” smartphone application.
